# The New Face of the Switch Flap in the Reconstruction of a Large Upper Eyelid Defect

**DOI:** 10.1155/2022/4159263

**Published:** 2022-02-12

**Authors:** Biljana Kuzmanović Elabjer, Mladen Bušić, Daliborka Miletić, Andrej Pleše, Mirjana Bjeloš

**Affiliations:** ^1^Faculty of Dental Medicine and Health Care Osijek, University Josip Juraj Strossmayer in Osijek, Croatia; ^2^Faculty of Medicine Osijek, University Josip Juraj Strossmayer in Osijek, Croatia; ^3^University Eye Clinic-WHO Collaborating Center, University Hospital “Sveti Duh”, Zagreb, Croatia

## Abstract

Reconstruction of a large defect after the removal of a massive malignant upper lid tumor is still a challenge in oculoplastic surgery. Our method of choice is Mustardé switch flap. Due to the lack of Mohs micrographic surgery and frozen section technique as well as waiting time of two weeks for histopathological results, we made modifications enabling us to reexcise in case of positive margins: the width of the pedicle of the flap was 7 mm allowing the length of the flap to be increased if needed, the lids were closed with temporary lateral tarsorrhaphy to protect the eye, and the lower lid is finally reconstructed in the second stage of the procedure. In three patients with malignant upper lid tumors, this method of reconstruction proved to be safe and effective with favorable long-term results.

## 1. Introduction

Mustardé switch flap for the upper lid defects, described in 1971, is still a state-of-the-art technique for achieving upper lid architecture, curve, eyelashes, and function, all in one [1, 2]. The major advantage to the other two-stage lid-sharing technique (Cutler-Beard procedure) is that the flap is divided in just two weeks as recommended by the author himself or as soon as seven days [1, 3]. The donor site of the lower lid is reconstructed either during the primary or secondary procedure [2–5]. Mohs micrographic surgery is unavailable in our institution, and histopathological results are obtained as late as two weeks. The upper lid defects are closed with Mustardé switch flap combined with temporary tarsorrhaphy and without any additional reconstruction until the histopathological report of either clear or infiltrating edges indicates further procedures. Thus, the final reconstruction of the upper and the lower eyelid is executed in the second stage of the procedure.

We present three patients with extensive upper lid tumors, reconstructed with our modified Mustardé switch flap achieving satisfactory functional and cosmetic results.

## 2. Case Presentation

### 2.1. Case No. 1

**Figure 1:**
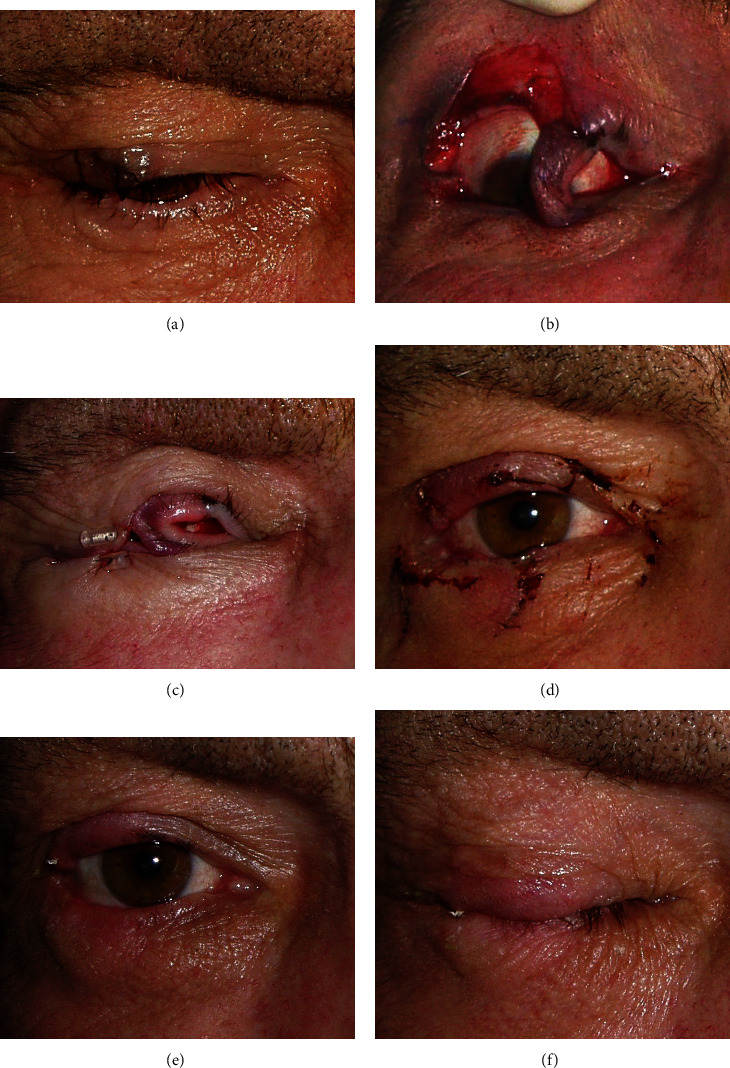
(a) A large nodular tumor with telangiectatic surface vessels involving the lateral half of the right upper lid. (b) The flap with a nasally oriented vascular pedicle. (c) The bolster of the lateral temporary tarsorrhaphy in place. (d) The reconstruction of the lower lid at the second stage of the procedure. (e) A satisfactory cosmetic appearance of both lids. (f) The full lid closure.

A 53-year-old male patient was referred to our Oculoplastic Unit for a large tumor involving the lateral half of the right upper lid (Figure 1(a)).

The procedure was performed under local anesthesia. According to the guidelines for well-defined lid tumors of our clinic and the literature [6], the tumor was removed with two millimeters of safety margins, creating a larger than half but not more than three-quarters full-thickness defect of the upper lid (Figure 1(b)). The flap with a nasally oriented vascular pedicle, 7 mm high, and half the length of the lower lid was turned up into the upper lid and hinged at the medial edge of the defect with 6/0 absorbable sutures and one 7/0 nylon suture for the grey line [7]. The aponeurosis of the levator muscle was sutured at the upper border of the flipped tarsal plate. Laterally, the lids were closed with temporary tarsorrhaphy (Figure 1(c)). After marking the adequate size of the tarsorrhaphy, a 6-0 single prolene suture was passed through the lids in a horizontal mattress fashion, 4 mm from the lid margin through the skin and muscle staying above the tarsal plate and through the grey line. To protect the skin, we used a bolster made of a vitrectomy cutter tube. The margins of the excised tumor were marked with different vital dyes before it was sent to histopathology. The second stage of the procedure was done two weeks after histopathological confirmation of a basal cell carcinoma with clear margins. The vascular pedicle was divided, and the lower lid was reconstructed using a tarso-conjunctival graft from the contralateral upper lid and a local advancement skin-muscle flap (Figure 1(d)) [8]. Good function and satisfactory cosmetic appearance of both lids were achieved (Figures 1(e) and 1(f)).

### 2.2. Case No. 2

**Figure 2:**
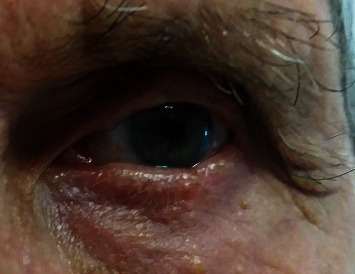
Clinical outcome 6 months after the surgery in Case No. 2.

Years-long history of a recurrent chalazion in the 70-year-old male patient with a lobular lesion involving one-quarter of the left upper lid were the reasons for the referral to our clinic. Due to the clinical aspect of the tumor suspicious of sebaceous cell carcinoma, the surgical excision included four-millimeter safety margins [9]. The primary surgery was performed in the same manner as in Case No. 1. Unfortunately, the sebaceous cell carcinoma was confirmed on histopathology 10 days after the surgery, with the tumor cells found at the medial edge, that is, the hinge side. Immediately, additional resection was carried out. The lids were divided as well as the hinge of the flap, and three millimeters of tissue at the medial edge of the primary defect was excised. The length of the flap was increased accordingly and sutured in the same fashion as in the primary surgery. The secondary surgery ended up with yet another temporary lateral tarsorrhaphy. Only when the excised tissue from the second surgery came free of the tumor after 9 days, the flap was divided, and the reconstruction of the lids was carried out and resulted in satisfactory function and cosmesis (Figure 2).

### 2.3. Case No. 3

**Figure 3:**
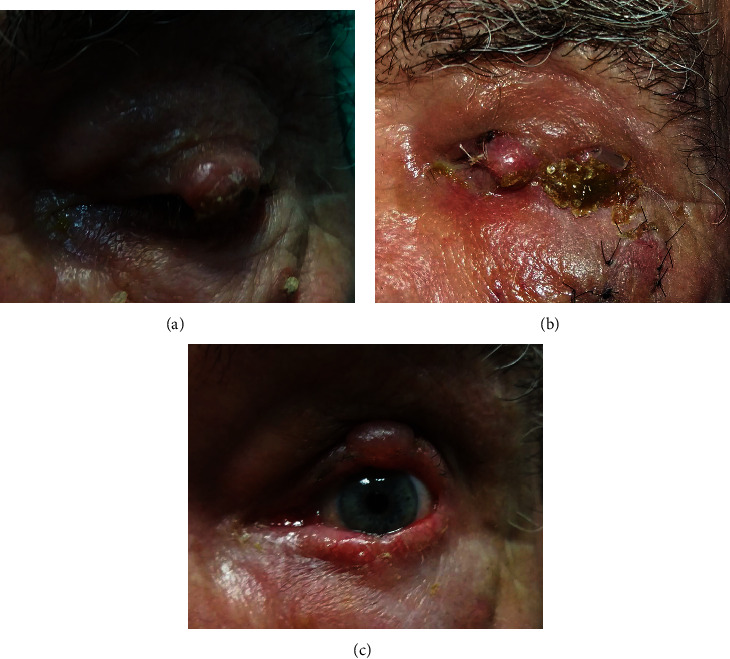
(a) An ulcerated tumor of the lateral half of the upper lid. Note the keratotic plaque at the right bottom of the photo. (b) The flap just before the pedicle separation. The nylon sutures of the modified rhomboid flap used for the reconstruction after the keratosis removal still in place. (c) A prolonged Mustardé switch flap edema.

An 80-year-old male patient presented with an ulcerated tumor of the lateral half of the upper lid (Figure 3(a)). The tumor removal and the first stage of reconstruction were conducted the same as in Case 1, except for setting the pedicle at the lateral end of the flap (Figure 3(b)).

After ten days, the histopathology proved squamous cell carcinoma with the margins free of the tumor, and the pedicle was divided. Lateral canthotomy and direct closure were sufficient for the lower lid reconstruction.

## 3. Discussion

Defects of the upper lid after excision of a malignant tumor, without the possibility of Mohs micrographic surgery, require creativity in combining the tissue to cover the eye and to still keep an open possibility to re-excise the edges in up to two weeks postoperatively. Our modification of the Mustardé switch flap enables exactly that the eye is covered and protected, and the lids are not primarily reconstructed so that the flap can be enlarged if needed. The length of the flap may be increased due to the width of the pedicle of the flap being 7 mm, that is, larger than originally recommended 6 mm for large defects [1]. Following the rule of Tyers and Collin, in a large medial remnant of the lid, the pedicle was sited medially; otherwise, it was hinged at the lateral edge of the flap [10]. The only issue we had with the traditional Mustardé switch flap was the risk of histopathological confirmation of the residual tumor and the necessity to cut out the pedicle of the flap. That was the scenario of our Case No. 2: we needed to start the flap all over again. In our opinion, the 7 mm-width-pedicle of the flap enables adjustments of the flap length and buys the time to wait for clear edges. Complications in healing after the lower lid reconstruction were not encountered even three weeks after the primary flap creation. Temporary lateral tarsorrhaphy was used to prevent corneal exposure for up to two weeks before the pedicle was cut. We used our modification of Collin's technique [7]: there was no incision of the grey line nor excision of the conjunctiva from the lid margin posterior to the grey line. This was particularly important in our modification of the Mustardé switch flap because after the tarsorrhaphy was undone the lids were easily separated with undamaged lid margin. Nevertheless, the lids were tightly closed for the period of the flap healing that lasted up to 19 days in our patients without any corneal problems.

The only complication we experienced was a prolonged Mustardé switch flap edema in Case No. 3 (Figure 3(c)) that resolved in four weeks. Mustardé emphasized that it may take several weeks for the eyelid swelling to subside following the flap [1]. The technique involves intense modification in the eyelid tissue's direction, with rotation from inferior to the superior eyelid, disfavoring lymphatic drainage. As oppose to the other two patients, Case No. 3 had the pedicle hinged at the lateral end of the flap where the outer canthus branch of a lymph-collecting vessel could have been surgically altered.

In his paper describing the switch flap in the treatment of malignant tumors of the upper lid, Mustardé always reconstructed the lower lid in the primary stage of the procedure [1]. Our modification thus opens the possibility of increasing the size of the Mustardé switch flap in case of need for additional excision following the findings on the histopathologic specimen. This serves well in settings where neither Mohs micrographic surgery nor frozen section is available and if histopathological results are not feasible in time.

## References

[1] Mustardé J. C. (1971). Surgical treatment of malignant tumours of the upper lid. *Chirurgia Plastica*.

[2] Mustardé J. C. (1981). Major reconstruction of the eyelids: functional and aesthetic considerations. *Clinics in Plastic Surgery*.

[3] Uemura T., Yanai T., Yasuta M., Kawano H., Ishihara Y., Kikuchi M. (2016). Switch flap for upper eyelid reconstruction-how soon should the flap be divided?. *Plastic and Reconstructive Surgery. Global Open*.

[4] Yamashita K., Yotsuyanagi T., Sugai A. (2020). Full-thickness total upper eyelid reconstruction with a lid switch flap and a reverse superficial temporal artery flap. *Journal of Plastic, Reconstructive & Aesthetic Surgery*.

[5] Stafanous S. N. (2007). The switch flap in eyelid reconstruction. *Orbit*.

[6] Quazi S. J., Aslam N., Saleem H., Rahman J., Khan S. (2020). Surgical margin of excision in basal cell carcinoma: a systematic review of literature: A Systematic Review of Literature. *Cureus*.

[7] Collin J. R. O. (1989). *A Manual of Systematic Eyelid Surgery*.

[8] Kuzmanović Elabjer B., Petrinović-Dorešić J., Bušić M., Elabjer E., Kaštelan S. (2007). Retrospective analysis of reconstruction techniques after periocular basalioma excision. *Collegium Antropologicum*.

[9] Yunoki T., Nakamura Y., Fuchizawa C., Hayashi A. (2017). Reconstructive surgery of the upper eyelid using the residual tarsus after excision of sebaceous gland carcinoma. *Case Reports in Ophthalmology*.

[10] Tyers A. G., Collin J. R. O. (2001). *Colour Atlas of Ophthalmic Plastic Surgery*.

